# Effectiveness of etilefrine regimen for chylothorax after esophagectomy with thoracic duct resection

**DOI:** 10.1007/s10388-017-0592-6

**Published:** 2017-09-14

**Authors:** Yu Ohkura, Masaki Ueno, Toshiro Iizuka, Harushi Udagawa

**Affiliations:** 1 Department of Gastroenterological Surgery, Toranomon Hospital, and Okinaka Memorial Institute for Medical Research, 2-2-2 Toranomon, Minato-ku, Tokyo, 105-8470 Japan; 2 Department of Gastroenterology, Toranomon Hospital, and Okinaka Memorial Institute for Medical Research, Tokyo, Japan

**Keywords:** Chylothorax, Etilefrine, Octreotide, Pleural adhesion, Esophagectomy

## Abstract

**Background:**

Management of postoperative chylothorax generally involves nutritional regimens as well as pharmacological and surgical therapies, but a clear consensus has yet to be reached.

**Methods:**

Retrospective review of 371 patients who underwent esophagectomy for esophageal cancer was performed. They were patients with squamous cell carcinoma or adenocarcinoma of the esophagus including Siewert type I/II tumor of the esophagogastric junction who underwent subtotal esophagectomy. Of these patients, 19 patients who were diagnosed with chylothorax as a postoperative complication were enrolled in this study.

**Results:**

Conservative treatment achieved cure in 16 patients among 19 patients. The duration of chylothorax tended to be longer in the no-etilefrine group (*n* = 5) than in the etilefrine group (*n* = 11) (27.8 vs. 11.6 days; *p* = 0.078). The 14 patients among 19 patients resected the thoracic duct. Etilefrine was used in 12 of these 14 patients. Among these 12 patients, 3 required surgical treatment and the remaining 9 patients were cured with conservative treatment. The duration of chylothorax was shorter in the conservative treatment group than in the surgical treatment group (11.9 vs. 36.3 days; *p* = 0.052). In addition, with the use of etilefrine as adjuvant therapy, cure was achieved in 9 patients (75%) without surgical intervention.

**Conclusions:**

The findings of this study suggest that when used concurrently with conventional treatments, etilefrine facilitates early chest tube removal. In addition, post-thoracic duct resection chylothorax, which frequently requires surgical treatment because of the general less effectiveness of conservative treatments, showed high successful rate (75%) to etilefrine treatment.

## Introduction

Postoperative chylothorax after esophagectomy occurs relatively infrequently, in about 2–9% of patients [1–3]. Management of postoperative chylothorax generally involves nutritional regimens as well as pharmacological and surgical therapies, but a clear consensus has yet to be reached [4]. Previous reports have noted the difficulty of using only medical management consisting of a nutritional regimen, etilefrine, octreotide, and picibanil (OK-432) for patients who have undergone thoracic duct resection, and that these therapies for chylothorax were indicated only for those patients in whom the thoracic duct was preserved [5–8]. However, if these therapies could be successful even in cases of thoracic duct resection, medical management alone would be advantageous because the patient would not need to undergo reoperation which might be associated with complications. In addition, it may be difficult to identify the site of leakage during surgery.

In this study, we investigated the usefulness of an etilefrine regimen to broaden the medical treatment options for postoperative chylothorax after esophagectomy with resection of the thoracic duct.

## Methods

### Study population

This single-center retrospective study was conducted to evaluate the effectiveness of therapy with an etilefrine regimen for chylothorax after esophagectomy. A total of 371 consecutive patients with esophageal cancer were identified from a prospectively constructed database at the Department of Gastroenterological Surgery, Toranomon Hospital between January 2011 and February 2017. They were patients with squamous cell carcinoma or adenocarcinoma of the esophagus including Siewert type I, II tumor of the esophagogastric junction who underwent subtotal esophagectomy. Of these 371 patients, 19 patients who were diagnosed with chylothorax as a postoperative complication were enrolled in this study. We investigated the clinical effectiveness of etilefrine for the management of postoperative chylothorax following esophagectomy particularly among patients with resection of the thoracic duct. Disease was staged according to the UICC TNM grading system, 7th edition [9]. We graded all postoperative complications based on the Clavien–Dindo classification [10], and grade ≥2 events were documented as complications. This study was conducted with approval from the Institutional Review Board of Toranomon Hospital.

### Operative procedure for esophagectomy

We perform esophagectomy with 2- or 3-field lymph node dissection depending on the degree of progression and surgical risk involved. The operative thoracic approach is by video-assisted thoracoscopic surgery or thoracotomy, and the abdominal approach is hand-assisted laparoscopic surgery or open laparotomy depending on individual cases. The thoracoscopic approach involves insertion of 5- or 12-mm ports through the second and fourth intercostal spaces on the anterior axillary line (assistant’s ports) and 11-mm ports through the fifth intercostal space at the mid-axillary line (camera port) and in the fourth (5 mm) and sixth (11 mm) intercostal spaces on the posterior axillary line (operator’s ports). We preserved the thoracic duct in cases with clinical stage (cStage) I and performed resection in cases with cStage ≥II based on the UICC TNM grading system, 7th edition [9]. The reconstruction technique was either gastric tube reconstruction, or ileocolon reconstruction.

### Definition of chylothorax

Chylothorax was suspected in the presence of excessive (>800 mL/day) chest drain output or if the color of chest drainage fluid turn milky white after tube feeding or oral ingestion. The pleural effusion was checked macroscopically to determine if we suspected. We made a diagnosis of chylothorax according to the criteria; the pleural fluid triglycerides (TG) >110 mg/dL, the ratio of pleural fluid TG to serum TG >1 and/or we made a confirmation of chylomicrons in the pleural drainage when the value of pleural fluid TG 50–110 mg/dL [11]. The duration of chylothorax was defined as days from the diagnosis of chylothorax to removal of the chest tube placed for the treatment of chylothorax.

### Management of chylothorax

Generally, postoperative chylothorax is managed either conservatively or surgically. The protocol followed in our hospital and in this study involves the application of a nutritional approach first, with patients receiving total parenteral nutrition. They are started on octreotide (300 μg/day) by continuous subcutaneous infusion and/or etilefrine (120 mg/day) by intravenous injection concurrently. If there is progressive resolution of the chylous pleural effusion with this treatment, and the effluent is approximately less than 50–100 mL/day, we perform pleurodesis with OK-432. After that, the patient made satisfactory progress and resumed oral food intake. If the effluent keep approximately less than 50–100 mL/day after the oral food intake started, the thoracotomy tube was removed. If conservative treatment fails (ex. in the presence of excessive (>400–500 mL/day) chest drain output), we then consider more invasive treatment such as lymphangiography, thoracic duct embolization, or thoracic duct ligation.

### Statistical analysis

All analyses were performed using the Statistical Package for the Social Sciences (SPSS) software version 19.0J for Windows (SPSS Inc., Chicago, IL). Pearson’s Chi-squared test, Fisher’s exact test, or the Mann–Whitney *U* test was used for intergroup comparisons, as appropriate. Statistical significance was set at *p* < 0.05.

## Results

### Outcomes of treatment for postoperative chylothorax (Fig. 1)

Of the 371 patients who underwent surgery for esophageal cancer, 19 patients (5.1%) developed postoperative chylothorax, for which preservation (*n* = 5) or resection (*n* = 14) of the thoracic duct was performed. Cure was achieved with conservative treatment in all 5 patients who had undergone thoracic duct preservation. The treatment results of 5 patients with thoracic duct preservation were two patients received etilefrine and octreotide regimen, another two patients received only nutrition approach and one patient started on octreotide only. In contrast, additional surgical treatment (thoracic duct ligation) was performed in 3 of the 14 patients who had undergone thoracic duct resection (TDR) because conservative treatment was insufficient to achieve cure. Thus, conservative treatment achieved cure in 16 patients among 19 patients with postoperative chylothorax.

**Fig. 1:**
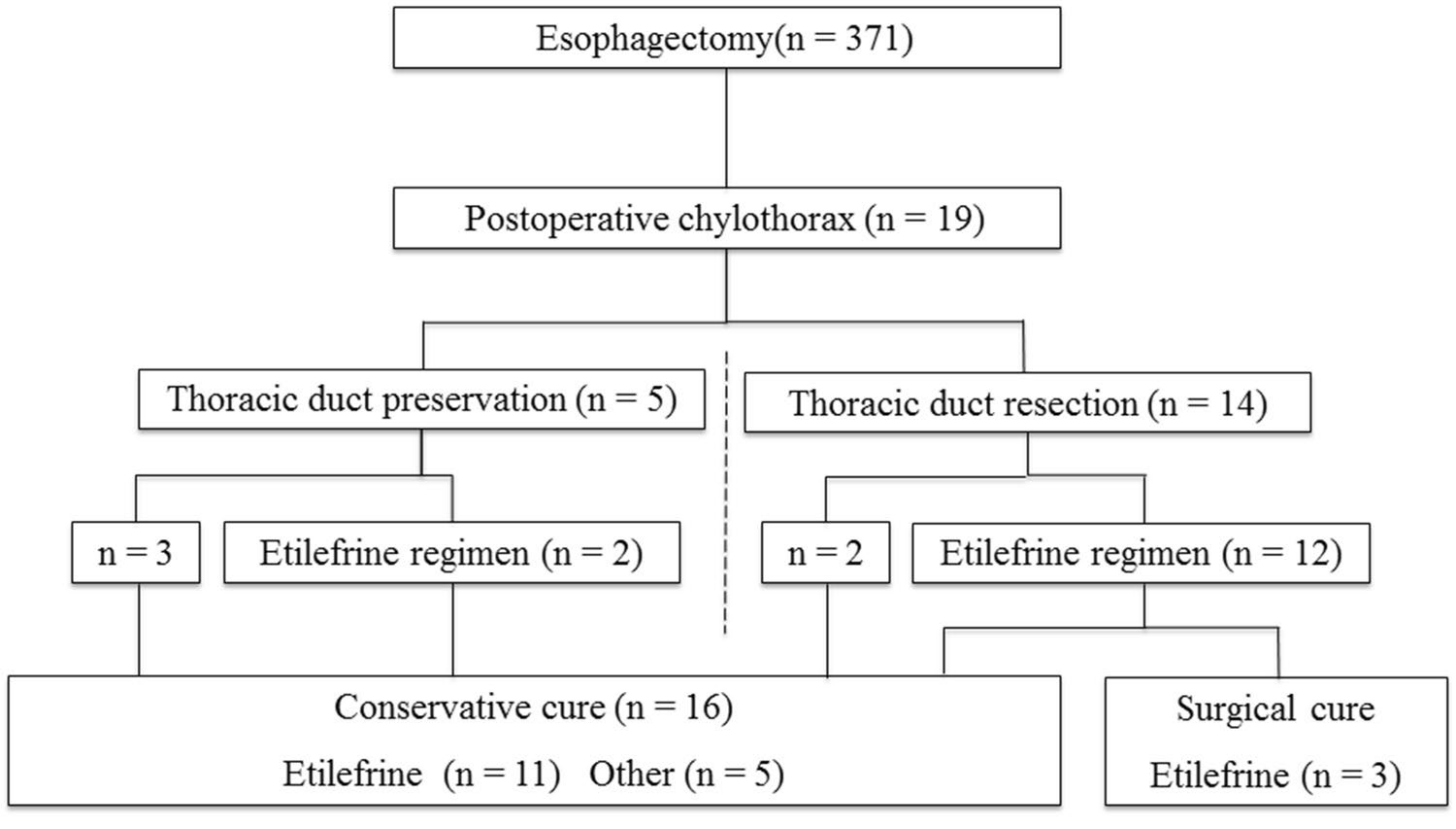
Outcomes of treatment for postoperative chylothorax

### Effectiveness of etilefrine treatment for postoperative chylothorax

Table 1 shows the background characteristics and surgical findings of 16 patients in whom cure was achieved after conservative treatment with (*n* = 11) and without (*n* = 5) adjuvant etilefrine treatment (see Fig. 1). Among patient background characteristics and intraoperative findings, no significant difference was found between age, sex, body mass index, clinical stage, tumor localization, extent of lymph node dissection, operative approach, resection/preservation of the thoracic duct, organ reconstruction, duration of the operation, or blood loss. The duration of chylothorax tended to be longer in the no-etilefrine group than in the etilefrine group, but no significant difference between the two groups (27.8 vs. 11.6 days, respectively; *p* = 0.078).

**Table 1:** Clinicopathological characteristics of the 16 patients in whom conservative cure of chylothorax was achieved

	Etilefrine regimen (*n* = 11)	Other (*n* = 5)	*p* value
Age: median (range)	62.2 (42–76)	63.6 (56–74)	0.910
Sex			0.513
Male	7	4	
Female	4	1	
Body mass index (kg/m^2^)	20.1 (14.7–26.0)	21.7 (17.4–26.3)	0.396
ASA			0.785
1	2	1	
2	8	4	
3–	1	0	
cStage (7th)			0.451
I (IA, IB)	4	2	
II (IIA, IIB)	1	2	
III (IIIA, IIIB)	5	0	
IVA	1	1	
Tumor localization			0.466
Ut	2	0	
Mt	5	4	
Lt	2	0	
Ae	0	0	
EGJ	2	1	
Lymphadenectomy			0.513
D2	4	1	
D3	7	4	
Operative approach (thoracic)			0.106
Open	3	4	
VATS	8	1	
Operative approach (abdominal)			0.210
Open	3	3	
HALS	8	2	
Thoracic duct			0.245
Preservation	2	3	
Resection	9	2	
Reconstruction organ			0.839
Gastric tube	6	3	
Ileocolon	5	2	
Duration of operation (min)	541 (358–702)	495 (350–631)	0.282
Amount of blood loss (mL)	496 (80–1413)	689 (285–1068)	0.157
Duration of chylothorax (days)	11.6 (2–31)	27.8 (6–74)	0.078

All figures followed by parentheses indicate a range unless otherwise stated

### Effectiveness of etilefrine treatment for postoperative chylothorax after thoracic duct resection

Etilefrine was used in 12 of 14 patients with post-TDR chylothorax. Among these 12 patients, 3 required surgical treatment and the remaining 9 patients were cured with conservative treatment. The background characteristics of patients had no significant differences between the groups. The duration of chylothorax was shorter in the conservative treatment group than in the surgical treatment group, but no significant difference between the two groups (11.9 vs. 36.3 days, respectively; *p* = 0.052) (the data not shown).

Table 2 shows detailed information for the 12 patients who developed chylothorax after esophagectomy with TDR. With the use of etilefrine as adjuvant therapy, cure was achieved in 9 patients (75%) without surgical intervention.

**Table 2:** Characteristics of patients with chylothorax after esophagectomy with thoracic duct resection treated with etilefrine regimen

Case	Age (years)	Sex	Thoracic duct	Octreotide	Etilefrine	OK-432	Operation	Timing of chylothorax diagnosis (POD)	The amount of fluid at the beginning (mL)	The daily average amount of chyle (mL)	Duration of drugs use (days)	Timing of reoperation (POD)	Duration of chylothorax (days)
1	73	F	Resected	○	○	✗	✗	5	635	271	9	–	5
2	43	M	Resected	○	○	✗	✗	7	810	219.6	3	–	6
3	54	F	Resected	○	○	✗	✗	11	1350	740	16	–	2
4	77	M	Resected	○	○	✗	○	8	1090	392.3	19	40	34
5	67	M	Resected	○	○	✗	○	3	1530	284.4	44	61	58
6	53	M	Resected	○	○	○	○	31	1053	426.8	17	48	17
7	65	F	Resected	○	○	○	✗	10	450	185.9	9	–	9
8	42	M	Resected	○	○	○	✗	12	200	124.5	6	–	8
9	69	M	Resected	○	○	○	✗	77	1730	977.5	12	–	18
10	67	M	Resected	○	○	○	✗	10	1140	198.5	30	–	31
11	68	F	Resected	○	○	○	✗	13	400	230	10	–	10
12	54	M	Resected	○	○	○	✗	32	2000	459.2	9	–	18

*POD* postoperative day

## Discussion

In this study, we have shown the effectiveness of etilefrine used in combination with conventional octreotide administration, pleurodesis, and nutritional therapy for the treatment of postoperative chylothorax following esophagectomy. Our findings suggest that when used concurrently with conventional treatments, etilefrine facilitates early chest tube removal. In addition, post-TDR chylothorax, which frequently requires surgical treatment because of the general less effectiveness of conservative treatments showed some favorable response to etilefrine treatment.

As reported previously, postoperative chylothorax is a relatively rare complication and is treated conventionally with nutritional therapy, drug therapy with octreotide, pleurodesis, and thoracic duct ligation. The use of etilefrine in chylothorax was reported for the first time by Guillem et al. [5]. Etilefrine has both α-adrenergic and β-adrenergic effects and is commonly used to treat conditions such as orthostatic hypotension and priapism. Guillem et al. explained that through its sympathomimetic effect, etilefrine induces contraction of the smooth muscles of the thoracic duct or main lymphatic duct, leading to a narrowed lumen, thereby decreasing chyle output. Furthermore, etilefrine is relatively safe because it rarely causes side effects such as headache, tachycardia, hypertension, and anxiety when used in proper dose. Therefore, we incorporated an etilefrine regimen for treating postoperative chylothorax into the protocol used in our hospital. As a result, we often can perform chest tube removal much earlier than we did before the incorporation of etilefrine.

Besides the present study, there have been hardly any reports showing the effectiveness of etilefrine in cases of chylothorax after esophagectomy with TDR [12]. In surgery for esophageal cancer, the thoracic duct is resected to ensure thorough removal of the lymph nodes around the thoracic duct, and the oncological effectiveness of TDR has been demonstrated [13, 14]. In performing TDR, we double-clip the thoracic duct above the diaphragm in patients with cStage II or more advanced esophageal cancer. Post-TDR chylothorax is mainly caused by major leakage from or near the ligation site and is difficult to treat conservatively. Conservative treatment alone is ordinally not sufficient to treat post-TDR chylothorax. Ojima et al. reported the effectiveness of etilefrine therapy in patients with preserved thoracic duct but not in those with post-TDR chylothorax [8]. In previous studies, conservative treatment (excluding thoracic duct embolization) had a success rate of 53.8% in postoperative chylothorax [3, 15–20] with or without TDR. In the present study, as many as 75% (9/12) of the patients with post-TDR chylothorax, in which conservative treatment has been reported to be ineffective, had a good outcome with the addition of etilefrine regimen. In other words, our findings suggest the effectiveness of etilefrine as a novel treatment for post-TDR chylothorax, which is conventionally considered intractable. The present study also shows that it took a relatively long time to achieve cure in surgically treated group because of the preceding conservative treatment. Therefore, when conservative treatment does not reduce chyle output in patients with post-TDR chylothorax, surgery should be considered as soon as possible to achieve cure in the early stage of the disease and to reduce patient burden. We think that the timing of reoperation is about 3–4 weeks after the conservative treatment was started.

The major limitations of our study were its single-center retrospective design and the small number of patients examined. Accumulation of cases would allow more precise analysis, which might further demonstrate the effectiveness of this treatment. A multicenter study with a larger number of cases is warranted.

## Conclusion

The findings of this study suggest the effectiveness of etilefrine in patients with chylothorax following esophagectomy. The drug was effective even in post-TDR chylothorax, an often intractable condition that is difficult to treat conservatively. However, when the effectiveness of etilefrine regimen is unexpectedly poor, it is important to switch from drug therapy to surgical treatment in the early stage of this complication.

## Abbreviation

TDR: Thoracic duct resection
